# Blood pressure in 3-year-old girls associates inversely with umbilical cord serum 25-hydroxyvitamin D: an Odense Child Cohort study

**DOI:** 10.1530/EC-18-0308

**Published:** 2018-10-04

**Authors:** Søs Dragsbæk Larsen, Christine Dalgård, Mathilde Egelund Christensen, Sine Lykkedegn, Louise Bjørkholt Andersen, Marianne Andersen, Dorte Glintborg, Henrik Thybo Christesen

**Affiliations:** 1 Department of Clinical Research, Faculty of Health Sciences, University of Southern Denmark, Odense, Denmark; 2 Department of Public Health, Environmental Medicine, University of Southern Denmark, Odense, Denmark; 3 Hans Christian Andersen Children’s Hospital, Odense University Hospital, Odense, Denmark; 4 Department of Obstetrics and Gynecology, Herlev Hospital, Copenhagen, Denmark; 5 Department of Medical Endocrinology, Odense University Hospital, Odense, Denmark

**Keywords:** vitamin D, blood pressure, sex-dimorphism, children

## Abstract

**Background:**

Low foetal vitamin D status may be associated with higher blood pressure (BP) in later life.

**Objective:**

To examine whether serum 25-hydroxyvitamin D_2+3_ (s-25OHD) in cord and pregnancy associates with systolic and diastolic BP (SBP; DBP) in children up to 3 years of age.

**Design:**

Prospective, population-based cohort study.

**Methods:**

We included 1594 singletons from the Odense Child Cohort with available cord s-25OHD and BP data at median age 3.7 months (48% girls), 18.9 months (44% girls) or 3 years (48% girls). Maternal s-25OHD was also assessed at gestational ages 12 and 29 weeks. Multiple regression models were stratified by sex *a priori* and adjusted for maternal educational level, season of birth and child height, weight and age.

**Results:**

In 3-year-old girls, SBP decreased with −0.7 mmHg (95% CI −1.1; −0.3, *P* = 0.001) and DBP with −0.4 mmHg (95% CI −0.7; −0.1, *P* = 0.016) for every 10 nmol/L increase in cord s-25OHD in adjusted analyses. Moreover, the adjusted odds of having SBP >90th percentile were reduced by 30% for every 10 nmol/L increase in cord s-25OHD (*P* = 0.004) and by 64% for cord s-25OHD above the median 45.1 nmol/L (*P* = 0.02). Similar findings were observed between pregnancy s-25OHD and 3-year SBP, cord s-25OHD and SBP at 18.9 months, and cord s-25OHD and DBP at 3 years. No consistent associations were observed between s-25OHD and BP in boys.

**Conclusion:**

Cord s-25OHD was inversely associated with SBP and DBP in young girls, but not in boys. Higher vitamin D status in foetal life may modulate BP in young girls. The sex difference remains unexplained.

## Introduction

High blood pressure (BP) increases cardiovascular risk and constitutes one of the primary modifiable risk factors for cardiovascular disease (1). Elevated BP in childhood increases the risk of prehypertension and hypertension in adulthood (2, 3), making it essential to prevent high BP in children. Identification of modifiable, early risk factors is a key in designing initiatives preventing future cardio-vascular events. Recently, hypovitaminosis D, defined as serum 25-hydroxyvitamin D (s-25OHD) <50 nmol/L, was suggested to be a risk factor for cardiovascular events (4). The potential cardioprotective properties of vitamin D stem from its biological actions including its ability to inhibit vascular smooth muscle cell proliferation and potentially regulate the renin–angiotensin system (RAS) (5, 6, 7). An inverse relationship between BP and vitamin D levels in both children and adults is observed in some, but not all, studies (5, 8, 9).

Hypovitaminosis D during pregnancy is a common global phenomenon (10, 11). Increasing evidence suggests that insufficient vitamin D status during foetal life may increase the risk of several adverse health outcomes in later life, although the mechanisms remain to be fully elucidated (12).

Adverse events *in utero* and in early life may have programming effects on BP and cardiovascular health in later life (13). Foetal vitamin D status depends solely on the 25OHD concentrations of the mother (10). Studies examining the association between maternal 25OHD concentrations during pregnancy and BP and other cardiovascular disease risk factors in the offspring have shown inconsistent results (14, 15, 16). However, an inverse association between maternal 25OHD in pregnancy and offspring systolic BP at age 9.9 years has been observed, suggesting a possible programming effect of foetal vitamin D status on child BP (14). Moreover, the inverse association between maternal s-25OHD in pregnancy and offspring BP at age 20 years was seen in females only (17), which indicates a possible sex-specific effect.

Our objective was to investigate whether s-25OHD in cord blood and during pregnancy (median gestational age 12 and 29 weeks) was associated with offspring systolic and diastolic blood pressures (SBP/DBP) in children up to about 3 years of age. The study derived from Odense Child Cohort (OCC), a large prospective birth cohort, which has previously been described in detail (18).

## Subjects and methods

### Study population

**Figure 1:**
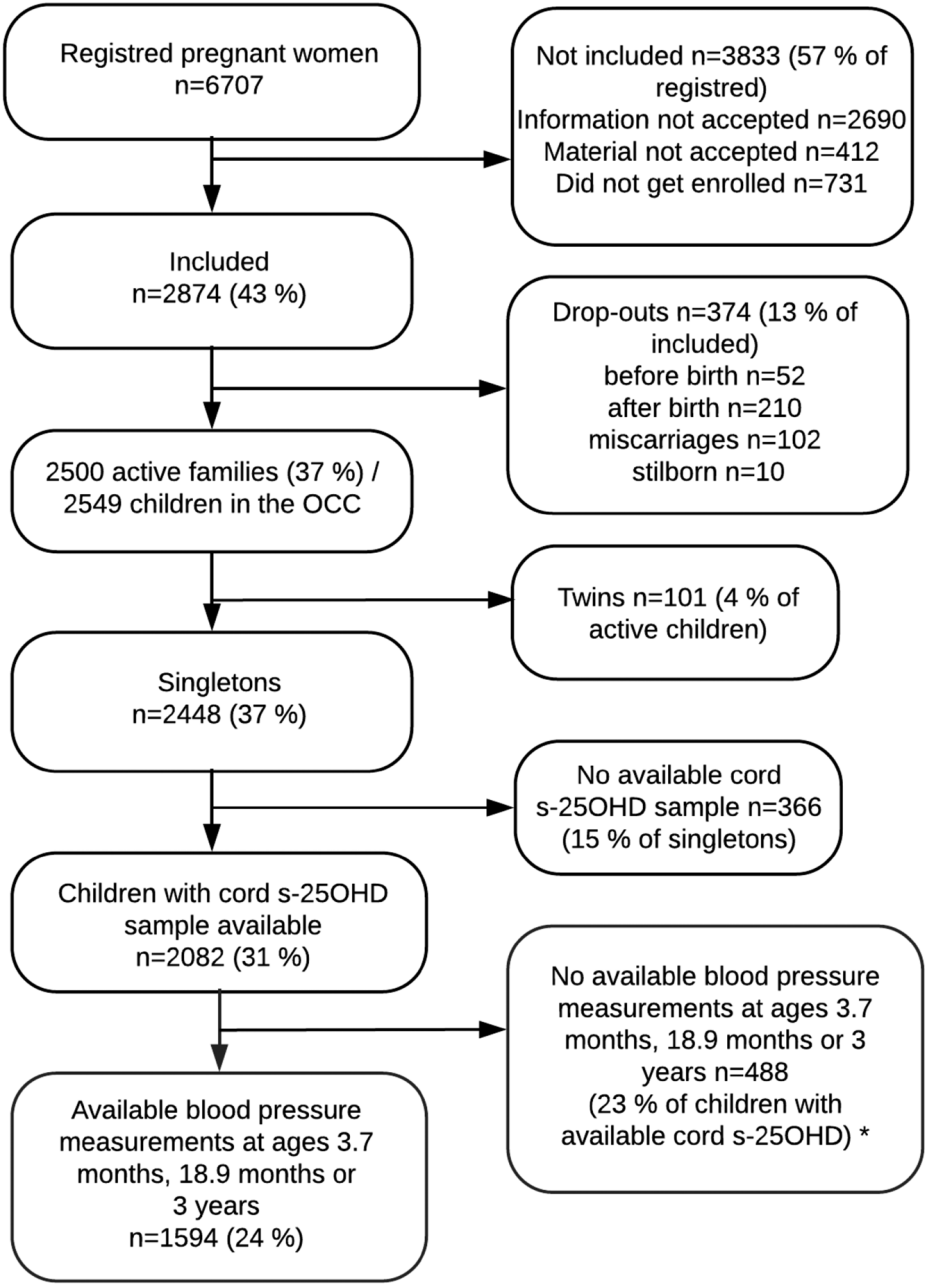
Flowchart of the study population. OCC, Odense Child Cohort; s-25OHD, serum 25-hydroxyvitamin D_2+3_. *Five measurements of systolic blood pressure and four of diastolic blood pressure were regarded an error of data entry and therefore coded as missing.

Participants were included from Odense Child Cohort (OCC), an on-going prospective, observational cohort consisting of mothers and their children. Inclusion criteria of OCC were residence in the Municipality of Odense and newly discovered pregnancy in the period of 1/1/2010–31/12/2012. Exclusion criteria were migration out of the municipality, miscarriage and stillbirth, resulting in 2549 active mother-children dyads. A more detailed description of the cohort has been published previously (18). Further exclusion criteria were multiple births, unavailable cord s-25OHD and missing measurements of BP at infant follow-up (Fig. 1).

We included 1594 children with measurements of cord s-25OHD and BP at a median and 25th–75th percentile [Q1; Q3] age of 3.7 [3.1; 4.4] months, 18.9 [18.3; 19.5] months and 36.0 [35.8; 36.6] months, respectively. SBP and DBP were measured in 1064 children (67%; 508 girls, 556 boys) at 3.7 months, 771 (48%; 341 girls, 430 boys) at 18.9 months and 1110 (70%; 530 girls, 580 boys) at 3 years.

### Exposure

Blood samples were drawn for analysis of s-25OHD from the umbilical cord and twice during pregnancy (median [Q1; Q3] gestational age 12 [10; 15] and 29 [28; 30] weeks). All samples were immediately processed and stored at −80°C until analysis. Concentrations of s-25OHD_2+3_ were assessed using liquid chromatography–tandem mass spectrometry (LC–MS/MS) as previously described, calibrated against National Institute of Standards and Technology, Standard Reference Material 972 with a CV of s-25OHD_2+3_ <7% (11). Cord s-25OHD was chosen as primary exposure. Maternal pregnancy blood samples were divided into early pregnancy (before week 20 + 0 days) and late pregnancy (week 20 + 0 onwards).

### Outcomes

Assessments of BP were performed at the age of 3 months, 18 months and 3 years by trained study staff. BP was measured in the left arm by a Welch Allyn device using a cuff of appropriate size. At 3 months of age, BP was measured with the child resting in a supine position; and at 18 months and 3 years, resting in the sitting position. Measurement of SBP at the age of 3 years was considered the primary outcome.

### Covariates

Maternal characteristics included the following self-reported data extracted from questionnaires: maternal skin tone on a modified Fitzpatrick’s Scale (19) (fair, darker white, brown or dark skin), maternal education (lower, intermediate or higher level), maternal sun exposure during pregnancy (never/rarely, sometimes, often, or most of the time), gestational weight gain and vitamin D supplementation during pregnancy (<10 µg/day or ≥10 µg/day). Data on maternal country of birth (western/non-western) were provided by the Municipality of Odense. Information on parity, smoking during pregnancy (smokers/non-smokers) and maternal pre-gestational BMI was extracted from self-reported data at the first antenatal visit. Maternal age was calculated at time of birth. Characteristics of the children including sex and gestational age (days) at birth were derived from medical files, while data of infant feeding (breastfed/non-breastfed, number of weeks of breastfeeding) and use of vitamin D supplementation (µg/day) were self-reported and extracted from questionnaires. Season of blood sampling was defined as either May–October or November–April, representing the seasons of high and low s-25OHD, respectively (11). Child weight was measured without clothing using a digital weight, and a stadiometer was used for the measures of height. Length was measured in a supine position at the age of 3 and 18 months; standing height at 3 years.

### Statistical analysis

Distribution of data was evaluated visually in qq-plots and presented as mean and standard deviations (SD) or median [Q1; Q3] where appropriate. Testing for differences between quartiles of cord s-25OHD was performed by ANOVA or Kruskal–Wallis test for continuous variables, and chi-square test was applied for categorical variables.

Multiple linear regression analyses were applied to test the associations between cord s-25OHD and BP. Moreover, the association of cord s-25OHD with BP >90th percentile was tested in multiple logistic regression analyses. Concentrations of cord s-25OHD were used as a continuous variable or categorised according to either median or quartiles. We conducted test for trend by evaluating the significance of s-25OHD grouped by quartiles. In addition, multiple linear regression models were performed with maternal s-25OHD in early and late pregnancy, respectively, as secondary exposure variables. Covariates were included in the multiple regression models if they were significantly related to BP, or if they were consistently used throughout the literature, and they were retained as potential confounders if they changed the main estimate (association between cord s-25OHD and BP) by >10%. The models were stratified by child sex *a priori*.

Model assumptions were checked for every linear model by evaluating the distribution of the studentized residuals in a qq-plot. Furthermore, logistic regression models were tested by Pearson goodness-of-fit test. Analyses were conducted using STATA 14.0 software (StataCorp). A significance level at *P* < 0.05 was chosen using two-sided tests.

The study was powered to detect a true change in SBP of 0.3 mmHg for every 10 nmol/L change in cord s-25OHD, given *n* = 1110, alfa = 0.05, beta = 0.80, cord s-25OHD SD 22 nmol/L and SBP SD = 8.

### Ethics

The women provided written informed consent to participate in the study approved by the Regional Scientific Ethical Committee for Southern Denmark, no S-20090130 and conducted in accordance with the Helsinki Declaration II. The study was also approved by the Danish Data protection Board, application no. 13/14088.

## Results

### Participants and variables

In the 1594 included children, median cord s-25OHD was 45.1 [30.6, 60.2] nmol/L, and 41.7% of the study population had levels of s-25OHD between 25.0 and 49.9 nmol/L (denoted vitamin D insufficiency), while 16.6% had levels below 25.0 nmol/L (denoted vitamin D deficiency). Characteristics of mothers and offspring according to quartiles of cord s-25OHD are shown in Table 1. Children with levels of cord s-25OHD in Q1 (<30.6 nmol/L) had mothers who were more often overweight or obese, smokers, of non-western ethnicity, and with higher parity, less sun exposure and lower vitamin D supplementation level compared to mothers of children with levels in the other quartiles, *P* < 0.01 for all. Children with cord s-25OHD <30.6 nmol/L had higher SBP, received less vitamin D supplementation per day and were more often born in the winter-spring period, *P* < 0.05 for all.

**Table 1:** Characteristics of the study population according to quartiles of cord s-25OHD.

	*N*	Quartiles of cord s-25OHD (nmol/L)				*P* Value
		<30.6	30.6–45.1	45.2–60.2	>60.2	
Maternal characteristics						
Gestational weight gain (kg)	675	15.0 (5.9)^a^	14.8 (5.6)	15.0 (5.5)	14.2 (5.2)	0.512
Maternal age (years)	1594	30.1 (4.5)	30.4 (4.4)	30.5 (4.5)	30.3 (4.4)	0.620
Pregestational BMI (kg/m^2^, %)						**<0.001**
Underweight: <18	51	2.8	1.3	3.8	5.0	
Normal weight: 18–24.9	1000	54.0	68.7	62.8	65.4	
Overweight: 25–29.9	370	25.6	21.3	24.6	21.3	
Obese: 30–35	120	11.3	5.3	24.6	6.8	
Severely obese: >35	53	6.3	3.5	6.8	1.5	
Smoker (%)						**0.005**
Yes	67	7.3	3.0	3.5	3.0	
No	1527	92.7	97.0	96.5	97.0	
Educational level (%)						0.595
Low	188	16.6	15.6	13.3	16.1	
Intermediate	741	58.8	58.3	61.5	63.6	
High	297	24.7	26.2	25.2	20.4	
Ethnicity (%)						**<0.001**
Western	1522	90.7	96.0	96.7	98.5	
Non-western	72	9.3	4.0	3.3	1.5	
Skin-type (%)						0.094
I/II White	239	23.0	20.1	17.9	16.3	
III Darker white	732	54.1	61.4	63.6	57.8	
IV Brown	248	21.0	17.3	17.6	25.2	
V/VI Dark brown	15	1.9	1.2	0.9	0.7	
Parity (%)						**0.002**
1	882	45.7	57.4	59.6	58.7	
2	542	40.5	32.1	30.1	32.6	
≥3	170	13.8	10.5	9.6	8.8	
Vitamin D supplementation (%)						**<0.001**
<10 µg/day	131	24.4	11.0	11.1	8.3	
≥10 µg/day	834	75.7	89.0	88.9	91.7	
Maternal sun exposure (%)						**0.007**
Never/rarely	24	2.6	2.2	1.6	1.4	
Sometimes	243	25.8	21.2	16.0	15.3	
Often	750	52.3	63.4	63.6	63.5	
Most of the time	219	19.4	13.2	18.8	19.9	
Paternal characteristics						
Paternal BMI (kg/m^2^, %)*						0.518
BMI <24.9	513	48.0	51.8	47.4	55.1	
Overweight: 25–29.9	397	38.6	38.9	41.4	37.1	
Obese: 30–35	86	10.6	7.8	9.7	5.7	
Severely obese: >35	20	2.9	1.6	1.5	2.0	
Child characteristics						
Height 3 years (months)	1099	0.97 (0.04)	0.97 (0.03)	0.97 (0.04)	0.97 (0.04)	0.220
Weight 3 years (kg)	1105	15.0 [13.8, 16.0]^b^	14.8 [13.6, 15.9]	14.9 [13.8, 16.0]	14.7 [13.6, 15.7]	0.264
BMI 3 years (kg/m^2^)	1084	15.8 [15.1, 16.5]	15.7 [15.0, 16.5]	15.8 [15.1, 16.5]	15.7 [15.1, 16.4]	0.674
SBP 3 years (mmHg)	1110	101 (8)	100 (7)	100 (7)	99 (7)	**0.018**
DBP 3 years (mmHg)	1110	63 (6)	63 (6)	62 (6)	62 (5)	0.232
Gestational age at birth (days)	1594	281 [275, 288]	281 [274, 287]	282 [275, 287]	282 [274, 288]	0.724
Child sex (%)						0.812
Boy	850	53.8	54.9	51.5	53.1	
Girl	744	46.2	45.1	48.5	46.9	
Season of blood sampling (%)						**<0.001**
May–October	846	30.2	44.4	61.8	75.9	
November–April	748	69.9	55.6	38.2	24.1	
Child age at 3 years examination (months)	1203	36.1 [35.8, 36.9]	36.0 [35.8, 36.5]	36.0 [35.8, 36.6]	36.0 [35.7, 36.4]	**0.014**
Duration of exclusive breastfeeding (weeks)	940	20 [15–24]	20 [16–24]	20 [16–24]	20 [16–24]	0.835
Vitamin D supplementation (µg/day)	836	3.1 [2.4–5.0]	5.0 [2.5–5.0]	4.8 [2.5–5.0]	5.0 [2.5–5.0]	**<0.001**

^a^Mean (s.d.) (all such numbers); ^b^median [Q1, Q3] (all such numbers); *the groups of BMI <18 and BMI 18–24.9 were merged because of *N* = 2 in BMI <18. Significant associations in bold.

The mean SBP/DBP at 3.7, 18.9 and 36 months were 102/61, 100/63 and 100/63 mmHg, respectively. For details, see Supplementary Table 1 (see section on supplementary data given at the end of this article).

### Cord s-25OHD and blood pressure association

Univariate linear regression analyses showed highly significant inverse associations between cord s-25OHD and SBP in 3-year-old girls (Table 2). Unadjusted associations between cord s-25OHD above vs below median and SBP in 3-year-old girls and DBP in 18.9 months and 3-year-old girls were also observed. Additionally, an inverse association between cord s-25OHD (continuous and above vs below median) and SBP >90th percentile in 3-year-old girls was found.

**Table 2:** Unadjusted associations of cord s-25OHD with systolic and diastolic blood pressure in girls in OCC.

	*N*	Continuous cord s-25OHD		Cord s-25OHD >50th percentile (ref. <50th percentile)	
		*β* (95% CI)	*P* Value	*β* (95% CI)	*P* Value
SBP					
3.7 months	508	0.01 (−0.04, 0.05)	0.854	0.23 (−1.94, 2.41)	0.832
18.9 months	341	−0.01 (−0.06, 0.04)	0.776	1.03 (−1.09, 3.14)	0.340
3 years	530	−0.05 (−0.08, −0.02)	**0.001**	−1.84 (−3.10, −0.59)	**0.004**
DBP					
3.7 months	508	0.003 (−0.04, 0.04)	0.875	−0.40 (−2.23, 1.44)	0.671
18.9 months	341	0.02 (−0.02, 0.06)	0.325	1.70 (0.07, 3.33)	**0.041**
3 years	530	−0.02 (−0.04, 0.004)	0.096	−1.12 (−2.11, −0.12)	**0.028**
		OR (95% CI)	*P* Value	OR (95% CI)	*P* Value
SBP >90th percentile (ref. SBP <90th p)					
3.7 months		1.00 (0.99, 1.02)	0.880	0.85 (0.42, 1.70)	0.637
18.9 months		0.99 (0.97, 1.01)	0.354	0.88 (0.42, 1.84)	0.732
3 years		0.97 (0.95, 0.99)	**<0.001**	0.44 (0.23, 0.85)	**0.015**
DBP >90th p (ref. DBP <90th p)					
3.7 months		1.00 (0.99, 1.02)	0.879	0.83 (0.44, 1.59)	0.581
18.9 months		1.00 (0.98, 1.02)	0.992	1.56 (0.67, 3.61)	0.301
3 years		0.99 (0.97, 1.00)	0.082	0.71 (0.40, 1.28)	0.254

Significant associations in bold.

CI, confidence interval; DBP, diastolic blood pressure; OR, odds ratio; ref., reference; s-25OHD, serum 25-hydroxyvitamin D_2+3_; SBP, systolic blood pressure.

**Figure 2:**
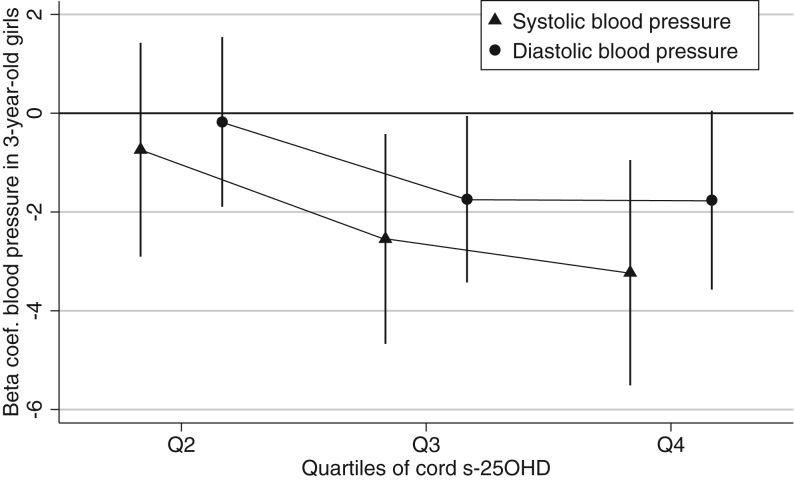
Adjusted associations of s-25OHD divided by quartiles with SBP and DBP in 3-year-old girls from Odense Child Cohort. The multiple linear regression model was stratified by sex and adjusted for maternal educational level, season of birth and child height, weight and age. Reference: Q1. Coef., Coefficient; Q, quartile; s-25OHD, serum 25-hydroxyvitamin D_2+3_.

Highly significant inverse associations were observed between cord s-25OHD and SBP and DBP in 3-year-old girls in multiple linear regression model adjusted for maternal educational level, season of birth and child height, weight and age (Table 3). The girls with cord s-25OHD levels above median had 2.5 mmHg lower SBP (*P* = 0.002) and 1.7 mmHg lower DBP (*P* = 0.009) than the girls with levels below the median. Significant trends toward lower SBP (*P* = 0.002) and DBP (*P* = 0.019) were observed with increasing cord s-25OHD quartiles (Fig. 2).

**Table 3:** Adjusted associations of cord s-25OHD with systolic and diastolic blood pressure in girls in OCC.

	*N*	Continuous cord s-25OHD		Cord s-25OHD >50th percentile (ref. <50th percentile)	
		*β* (95% CI)	*P* Value	*β* (95% CI)	*P* Value
SBP					
3.7 months	376	0.01 (−0.04, 0.07)	0.618	0.58 (−2.03, 3.18)	0.664
18.9 months	258	−0.02 (−0.09, 0.04)	0.476	−0.72 (−3.18, 1.73)	0.561
3 years	384	−0.07 (−0.11, −0.03)	**0.001**	−2.47 (−4.03, −0.88)	**0.002**
DBP					
3.7 months	376	0.013 (−0.04, 0.06)	0.610	−0.14 (−2.34, 2.06)	0.900
18.9 months	258	0.005 (−0.05, 0.06)	0.855	0.40 (−0.61, 1.50)	0.699
3 years	384	−0.04 (−0.07, −0.01)	**0.016**	−1.66 (−2.91, −0.41)	**0.009**
		OR (95% CI)	*P* Value	OR (95% CI)	*P* Value
SBP >90th percentile (ref. SBP <90th p)					
3.7 months		0.01 (0.99, 1.03)	0.365	1.16 (0.48, 2.80)	0.742
18.9 months		0.97 (0.95, 1.00)	**0.029**	0.33 (0.11, 0.93)	**0.036**
3 years		0.97 (0.95, 0.99)	**0.004**	0.36 (0.15, 0.85)	**0.020**
DBP >90th p (ref. DBP <90th p)					
3.7 months		1.00 (0.98, 1.02)	0.986	0.82 (0.37, 1.81)	0.626
18.9 months		1.00 (0.97, 1.02)	0.911	1.17 (0.42, 3.25)	0.760
3 years		0.98 (0.96, 1.00)	**0.045**	0.58 (0.28, 1.22)	0.154

All models were stratified by sex and adjusted for maternal educational level, season of birth and child height, weight and age. Significant associations in bold.

CI, confidence interval; DBP, diastolic blood pressure; OR, odds ratio; ref., reference; s-25OHD, serum 25-hydroxyvitamin D_2+3_; SBP, systolic blood pressure.

The adjusted odds of having SBP >90th percentile were reduced by 30% for every 10 nmol/L increase in cord s-25OHD at 18.9 months and 3 years in girls and by 20% for DBP in 3-year-old girls (Table 3). Likewise, the adjusted odds for having SBP >90th percentile were reduced by two thirds for 18.9-months- and 3-years-old girls with cord s-25OHD above the median of 45.1 nmol/L. No associations between s-25OHD and BP were detected at the age of 3.7 months.

In boys, no consistent crude or adjusted associations were observed between cord s-25OHD and BP at any age (Supplementary Tables 2 and 3). *Post hoc* testing of an interaction term between child sex and cord s-25OHD did not reach statistical significance in any of the analyses (data not shown).

### Pregnancy s-25OHD and blood pressure association

SBP was inversely associated with s-25OHD in early and late pregnancy in adjusted analyses in 3-year-old girls, whereas DBP was not associated with s-25OHD (Table 4). SBP/DBP at the age of 3.7 and 18.9 months was not associated with maternal s-25OHD sampled in early and late pregnancy (data not shown). Additionally, no association between maternal pregnancy s-25OHD concentrations and BP were seen in boys (data not shown). No *post hoc* interaction terms between child sex and pregnancy s-25OHD reached statistical significance (data not shown).

**Table 4:** Associations of cord s-25OHD and maternal pregnancy s-25OHD levels with systolic and diastolic blood pressure in girls aged 3 years.

	*N*	Adjusted model	*P*-Value
		*β* (95% CI)	
SBP 3 years			
Early pregnancy s-25OHD	214	−0.05 (−0.10, −0.003)	**0.038**
Late pregnancy s-25OHD	268	−0.06 (−0.09, −0.02)	**0.001**
Cord s-25OHD	384	−0.07 (−0.11, −0.03)	**0.001**
DBP 3 years			
Early pregnancy s-25OHD	214	−0.01 (−0.05, 0.03)	0.700
Late pregnancy s-25OHD	268	−0.02 (−0.05, 0.002)	0.066
Cord s-25OHD	384	−0.04 (−0.07, −0.01)	**0.016**

All models were stratified by sex and adjusted for maternal educational level, season of birth and child height, weight and age. Significant associations in bold.

CI, confidence interval; DBP, diastolic blood pressure; s-25OHD, serum 25-hydroxyvitamin D_2+3_; SBP, systolic blood pressure.

## Discussion

In this large Danish prospective cohort study, we found an inverse association between cord s-25OHD and SBP and DBP in 3-year-old girls with evidence of a dose–response relationship. No such consistent associations between s-25OHD and BP were found in boys.

The inverse association between cord s-25OHD and BP in girls was strongest at 3 years, but significant findings were already detectable at 18.9 months with reduced odds of having SBP >90th percentile with higher cord s-25OHD. Additionally, SBP in girls at three years was inversely associated with maternal s-25OHD in both early and late pregnancy. The strength of the inverse association seemed to increase with gestational age, indicating critical importance of sufficient levels of s-25OHD on SBP especially in late gestation and up to birth.

In concurrence with our study, a large observational study by Williams *et al.* found an inverse association between maternal s-25OHD in late pregnancy and offspring SBP measured at 9.9 years, however, the association was not present at 15.4 years (14). Moreover, no analyses stratified by offspring sex were performed. Smaller observational studies (15, 16), one with univariate analysis only (15) and one exclusively comparing the BP in children of deficient (s-25OHD <50 nmol/L) and non-deficient mothers in adjusted analyses (16), have shown no associations at age 5, 9 and 9.5 years. In agreement with the sex-differential findings from the present study, a Danish cohort study by Rytter *et al.* found a crude inverse association between maternal s-25OHD concentration during week 30 of gestation and offspring BP at age 20 years in girls only. However, the association did not reach statistically significance in adjusted analyses (17). Another observational study did not find an association between neonatal s-25OHD and BP at 35 years age (20), but no investigations of sex-specific patterns were performed regarding BP.

To the best of our knowledge, the association between vitamin D and BP in girls only has not been described previously with the exception of the indications of a possible sex-specific association in Rytter *et al*. (17). We present no biological explanation for the sex-differential pattern in our study. However, increasing evidence suggests that sex hormones might affect BP and that oestrogens may be a protective factor potentially via modulation of RAS (21, 22). Additionally, animal studies indicate that oestrogen may also have a protective effect on developmental programming of BP in females in young adulthood (23). We may speculate that vitamin D in pregnancy affects sex hormone patterns in favour of a protective effect on BP in girls via RAS. Sex-specific findings have previously been described regarding to the association between maternal s-25OHD and HDL cholesterol and percentage body fat in boys in the study by Krishnaverni *et al.* (16). Moreover, Christensen *et al.* identified an inverse association between late pregnancy and cord s-25OHD and lower leg length in boys (24).

In this study, we observed SBP and DBP lowered with 2.5 mmHg and 1.7 mmHg respectively when s-25OHD levels were above the median concentration in girls aged 3 years. We currently do not know the magnitude of the clinical and public health consequences of such BP differences in childhood, or whether this difference remains later in childhood and adolescence. However, studies show a strong association between childhood BP and BP later in life (3). Further studies into the consequences of childhood BP on cardiac health in later life are needed to fully understand the implications of our findings.

The major strengths of our study include the use of a large population-based birth cohort; longitudinal s-25OHD sampling and detailed anthropometric measures by trained study staff blinded for s-25OHD analysis; the use of s-25OHD, which is generally agreed to be the best marker for vitamin D status, with the gold standard LC–MS/MS method and robust statistical analysis. It is unknown whether the routine s-25OHD cut-offs are applicable in pregnancy and cord blood for non-skeletal health outcomes. Therefore, statistical analyses were performed primary by using median and quartiles analysis, and only secondarily by routine cut-off values.

Limitations included the observational nature of the study, a risk of selection bias, as women included in OCC have higher socio-economic status and are more frequently of ethnic Danish origin compared to the background population (18). Other limitations include utilization of self-reported data and the single measure of BP by Welch Allyn device, which, however, correlates reasonably well with auscultation and is preferred when measuring BP in newborns and infants because of the difficulty of auscultation in this age group (25).

### Conclusion

In this large, Danish cohort, we found an inverse association between cord s-25OHD and BP in 3-year-old girls, but not in boys. Higher vitamin D status in foetal life may modulate BP in young girls.

## Supplementary Material

Supporting Table 1

Supporting Table 2

Supporting Table 3

## Declaration of interest

The authors declare that there is no conflict of interest that could be perceived as prejudicing the impartiality of the research reported.

## Funding

This research did not receive any specific grant from any funding agency in the public, commercial or not-for-profit sector.
