# Supplementary material for: Blood pressure in 3-year-old girls associates inversely with umbilical cord serum 25-hydroxyvitamin D: an Odense Child Cohort study
Source: Endocr Connect. 2018 Oct 4;7(12):1236–44. doi: 10.1530/EC-18-0308 (PMC6240151; doi:10.1530/EC-18-0308)
Supplement: Supporting Table 1 [file EC-18-0308-t001.pdf]

SUPPLEMENTARY TABLE 1 Outcome characteristics of the children with available information on cord s-25OHD

	Total		Girls		Boys	
	n	Mean (SD)	n	Mean (SD)	n	Mean (SD)
SBP 3.7 months	1064	102 (14)	508	99 (12)	556	104 (15)
SBP 18.9 months	771	100 (10)	341	100 (10)	430	101 (9)
SBP 36.0 months	1110	100 (7)	530	100 (7)	580	100 (7)
DBP 3.7 months	1064	61 (11)	508	60 (10)	556	61 (12)
DBP 18.9 months	771	63 (8)	341	62 (8)	430	63 (8)
DBP 36.0 months	1110	63 (6)	530	63 (6)	580	62 (6)

Abbreviations: S-25OHD, serum 25-hydroxyvitamin D_{2+3} ; SBP, systolic blood pressure; DBP, diastolic blood pressure.
