# Supplementary material for: Blood pressure in 3-year-old girls associates inversely with umbilical cord serum 25-hydroxyvitamin D: an Odense Child Cohort study
Source: Endocr Connect. 2018 Oct 4;7(12):1236–44. doi: 10.1530/EC-18-0308 (PMC6240151; doi:10.1530/EC-18-0308)
Supplement: Supporting Table 2 [file EC-18-0308-t002.pdf]

SUPPLEMENTARY TABLE 2 Unadjusted associations of s-cord 25OHD with systolic and diastolic blood pressure in boys.

		Continuous cord s-25OHD		Cord s-25OHD>50th percentile (ref.<50th percentile)	
	N	β (95 % CI)	P-value	β (95 % CI)	P-value
SBP					
3.7 months	556	0.03 (-0.03, 0.08)	0.419	1.29 (-1.18, 3.75)	0.307
18.9 months	430	0.001 (-0.04, 0.04)	0.976	-0.36 (-2.14, 1.41)	0.687
3 years	580	-0.02 (-0.05, 0.01)	0.153	-0.62 (-1.80, 0.56)	0.302
DBP					
3.7 months	556	0.01 (-0.03, 0.06)	0.639	0.32 (-1.70, 2.33)	0.759
18.9 months	430	0.01 (-0.03, 0.05)	0.637	0.54 (-1.05, 2.13)	0.507
3 years	580	-0.01 (-0.03, 0.01)	0.347	-0.50 (-1.40, 0.41)	0.281
	N	OR (95 % CI)	P-value	OR (95 % CI)	P-value
SBP>90th percentile (ref. SBP<90th p)					
3.7 months		1.01 (0.99, 1.02)	0.417	1.35 (0.80, 2.25)	0.260
18.9 months		0.99 (0.98, 1.01)	0.331	0.68 (0.36, 1.28)	0.229
3 years		0.98 (0.97, 1.00)	**0.015**	0.65 (0.36, 1.18)	0.159
DBP>90th p (ref. DBP<90th p)					
3.7 months		1.00 (0.99, 1.01)	0.872	0.97 (0.56, 1.68)	0.917
18.9 months		1.00 (0.99, 1.02)	0.644	1.18 (0.64, 2.08)	0.593
3 years		0.99 (0.98, 1.01)	0.351	0.93 (0.51, 1.69)	0.815

Abbreviations: S-25OHD, serum 25-hydroxyvitamin D_{2+3}; SBP, systolic blood pressure; DBP, diastolic blood pressure; CI, confidence interval; ref., reference; OR, odds ratio. Significant associations in bold.
