# Supplementary material for: Blood pressure in 3-year-old girls associates inversely with umbilical cord serum 25-hydroxyvitamin D: an Odense Child Cohort study
Source: Endocr Connect. 2018 Oct 4;7(12):1236–44. doi: 10.1530/EC-18-0308 (PMC6240151; doi:10.1530/EC-18-0308)
Supplement: Supporting Table 3 [file EC-18-0308-t003.pdf]

*SUPPLEMENTARY TABLE 3* Adjusted associations of cord s-25OHD with systolic and diastolic blood pressure in boys.

| | | Continuous cord s-25OHD | | Cord s-25OHD>50$^{th}$ percentile (ref.<50$^{th}$ percentile) | |
|---|---|---|---|---|---|
| | N | $\beta$ (95 % CI) | P-value | $\beta$ (95 % CI) | P-value |
| SBP | | | | | |
| 3.7 months | 428 | 0.01 (-0.06, 0.07) | 0.811 | 0.70 (-2.04, 3.44) | 0.616 |
| 18.9 months | 342 | 0.02 (-0.03, 0.08) | 0.364 | -0.07 (-2.20, 2.07) | 0.952 |
| 3 years | 427 | -0.02 (-0.06, 0.02) | 0.259 | -0.75 (-2.26, 0.76) | 0.327 |
| DBP | | | | | |
| 3.7 months | 428 | -0.002 (-0.05, 0.05) | 0.945 | -0.33 (-2.52, 1.87) | 0.769 |
| 18.9 months | 342 | 0.02 (-0.02, 0.07) | 0.362 | 0.69 (-1.19, 2.56) | 0.471 |
| 3 years | 427 | -0.004 (-0.03, 0.02) | 0.780 | -0.27 (-1.41, 0.87) | 0.644 |
| | N | OR (95 % CI) | P-value | OR (95 % CI) | P-value |
| SBP>90$^{th}$ percentile (ref. SBP<90$^{th}$ p) | | | | | |
| 3.7 months | | 0.99 (0.98, 1.01) | 0.219 | 0.91 (0.47, 1.75) | 0.770 |
| 18.9 months | | 1.00 (0.98, 1.02) | 0.899 | 0.72 (0.32, 1.65) | 0.438 |
| 3 years | | 0.99 (0.97, 1.01) | 0.367 | 0.80 (0.38, 1.70) | 0.566 |
| DBP>90$^{th}$ p (ref. DBP<90$^{th}$ p) | | | | | |
| 3.7 months | | 1.00 (0.98, 1.02) | 0.916 | 0.87 (0.43, 1.76) | 0.699 |
| 18.9 months | | 1.01 (0.99, 1.03) | 0.237 | 1.48 (0.70, 3.11) | 0.307 |
| 3 years | | 1.00 (0.98, 1.02) | 0.832 | 1.37 (0.65, 2.90) | 0.154 |

Abbreviations: S-25OHD, serum 25-hydroxyvitamin $D_{2+3}$; SBP, systolic blood pressure; DBP, diastolic blood pressure; CI, confidence interval; ref., reference; OR, odds ratio. All models are stratified by sex and adjusted for maternal educational level, season of birth and child height, weight and age. Significant associations in bold.
